# A stumbling block or a stepping stone?

**DOI:** 10.1007/s12471-016-0813-y

**Published:** 2016-02-15

**Authors:** F. M. Zimmermann, L. R. Dekker

**Affiliations:** Department of Cardiology, Catharina Hospital Eindhoven, Eindhoven, The Netherlands

**Question**

**Fig. 1:**
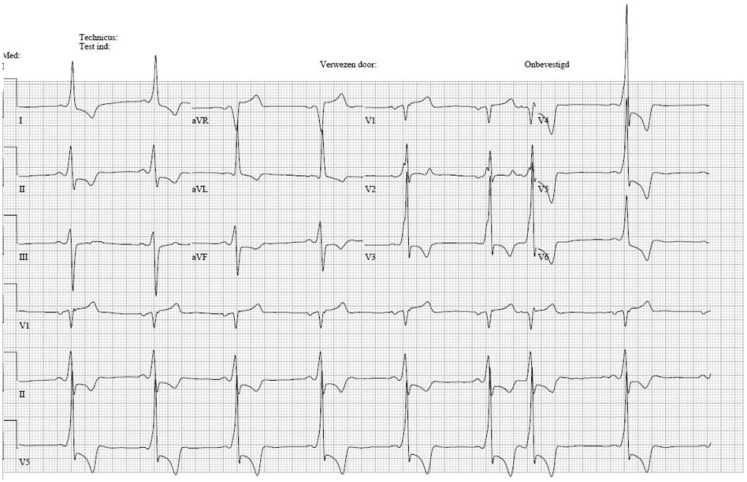
ECG at rest

**Fig. 2:**
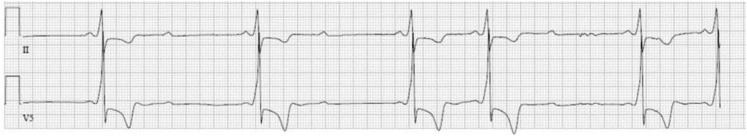
ECG in the emergency room

A 60-year-old man, known with Fabry disease, secondary left ventricular hypertrophy, and moderate aortic valve stenosis had a check-up in the outpatient clinic. An electrocardiogram (ECG) at rest (Fig. 1) showed sinus rhythm, a short PR interval, QRS interval of 120 ms with a sharp initial part suggestive of pre-excitation, as well as other unchanged abnormalities, including signs of left ventricular hypertrophy. In order to examine this possible pre-excitation suggestive of a right-sided, septal bypass an exercise test was performed. Unfortunately, the test was stopped after only 2 min due to exhaustion with a maximal heart rate of only 83/minute, without changes in QRS morphology. Because of a negative history of collapse or palpitations no further diagnostic tests were performed. Four months later, he was admitted because of a collapse. The ECG at presentation is shown in Fig. 2.

What is your diagnosis, and what does it tell you about the possible pre-excitation?

**Answer**

You will find the answer elsewhere in this issue.

